# *Toxoplasma gondii* in Wild Boars (*Sus scrofa*) in Germany: Serological Screening from Thuringia

**DOI:** 10.3390/ani14152148

**Published:** 2024-07-24

**Authors:** Zaida Rentería-Solís, Paul Deutschmann, Thomas W. Vahlenkamp, Kristin Heenemann

**Affiliations:** 1 Institute of Parasitology, Centre for Infection Medicine, Faculty of Veterinary Medicine, Leipzig University, 04103 Leipzig, Germany; 2 Albrecht-Daniel-Thaer Institute, Rudolf-Breitscheid-Str. 38, 04463 Groesspoesna, Germany; 3 Institute of Virology, Faculty of Veterinary Medicine, Leipzig University, An den Tierkliniken 29, 04103 Leipzig, Germany

**Keywords:** Apicomplexa, game meat, meat borne parasites, *Sus scrofa*, Zoonoses

## Abstract

**Simple Summary:**

Parasites can be transmitted from animals to humans by consumption of undercooked meat. One of these parasites is *Toxoplasma gondii*, a unicellular parasite that could cause severe disease in immunocompromised patients and foetal problems during pregnancy. *T. gondii* can be found in wild boar meat and be a source of infection for humans, particularly hunters and their inner circle of acquaintances. We tested serum samples of 42 free-ranging wild boars from Thuringia in central Germany during the hunting season of 2017/2018 and were able to detect antibodies against *T. gondii* in 18 of these animals (37.50%). The explicit prevalence of *T. gondii* specific antibodies points towards a risk of toxoplasmosis for people consuming game meat, particularly when it is not properly cooked. The hunting community as well as authorities should be aware of such possible exposures and infections. Additional studies among free-ranging wild boars would help to obtain more prevalence data from other areas of Germany and to better estimate the actual risk of *T. gondii* infection among the hunting community and game meat consumers’ interface.

**Abstract:**

Game meat is an important source of meat borne parasitic infections. Due to its omnivorous diet, the wild boar is an important host of zoonotic parasites such as *Toxoplasma gondii*. *T. gondii* can cause severe to fatal disease in immunosuppressed patients, as well as congenital disorders in foetus and neonates. Consumption of undercooked infected meat is a main source of *T. gondii* infection. Information about the risk of toxoplasmosis through game meat is scarce. We collected serum samples from 42 wild boars from the federal state of Thuringia (Germany) between December 2017 and February 2018. Identification of anti-*T. gondii* IgG antibodies was conducted using a commercial indirect ELISA kit. Seropositivity was confirmed in 18 of the 42 samples (37.50%). From these, the highest seroprevalence was found in adult animals. This study joins another single database from wild boars in Brandenburg. The necessity of a country-wide database regarding *T. gondii* prevalence in wild boar and other game meat is pivotal for a profound risk analysis with its consequential impact in future mean hygiene policies.

## 1. Introduction

Meat borne parasites are an often neglected source of zoonotic disease [1]. This disregard can be in part due to the current intensive livestock production methods, which are considered to have significantly reduced the presence of food borne parasites in the meat industry [2]. Intensive animal production sites could limit the access to potential hosts that facilitate the parasite’s completion of its life cycle. Game, conversely, is not submitted to the same anthropogenic stress and the main pressure for the parasite’s survival, including host infection, comes primarily from the ecological variations in the natural environment of its host(s) [3].

Game meat is, therefore, commonly found to be at higher risk of parasite contamination than livestock [4,5]. In Germany, its consumption amongst the general population is low in comparison with livestock meat. However, the intensive consumer, namely hunters, their family and acquaintance circle, consumes game meat around 60 times per year [4,6]. This is in part because it is considered a lean meat and source of proteins and other nutrients, like healthy fatty acids [7]. Game meat includes different species of wild animals, including cervids such as roe deer (*Capreolus capreolus*), lagomorphs like hares (*Lepus* spp.), or even omnivores like raccoons (*Procyon lotor*) [8].

An important omnivore regularly hunted for its meat is the wild boar (*Sus scrofa*). Wild boars are widely found in Eurasia and north Africa and have been introduced to the Americas and Oceania. They can adapt, not only to different ecosystems but also to the use of anthropogenic resources [9]. Their omnivore diet and adaptability make them a common host for several zoonotic meat borne parasites. Including helminths and their larval forms of importance in meat hygiene and public health such as *Trichinella* spp. [10], *Taenia* spp. [11], *Echinococcus* spp. [12], and *Alaria alata* [13].

A major zoonotic parasite found in wild boar meat is *Toxoplasma gondii*, an apicomplexan protozoon with global distribution. Around a quarter of the global human population is seropositive to *T. gondii* [14], with the highest seroprevalence found in Africa (61.40%) while 29.60% of the population of Europe is reported to be seropositive [14]. *T. gondii*’s final host are all members of the Felidae family in which the parasite can produce oocysts that are shed in the faeces [15]. Intermediate hosts, namely all warm-blooded vertebrates, can become infected by ingestion of oocysts in water, food or contaminated surfaces. After entering the intermediate, *T. gondii* will colonize different organs and finally develop into an encysted resting stage called bradyzoite. Bradyzoites can further reach cats and other intermediate hosts (including humans) by predation of infected prey, or by consumption of undercooked meat in the case of humans and domestic carnivores [16]. Toxoplasmosis in humans is of particular importance for immunosuppressed and pregnant patients. In the former, it can be manifested as a neurological disease mainly due to the reactivation of encysted bradyzoites; also in immunocompromised patients, multiple organs can be compromised when the infection is acute [17]. During pregnancy, congenital problems for the foetus or neonate are seldom but can occur in the form of neurological disorders such as hydrocephalous or microcephalus [18].

**Figure 1:**
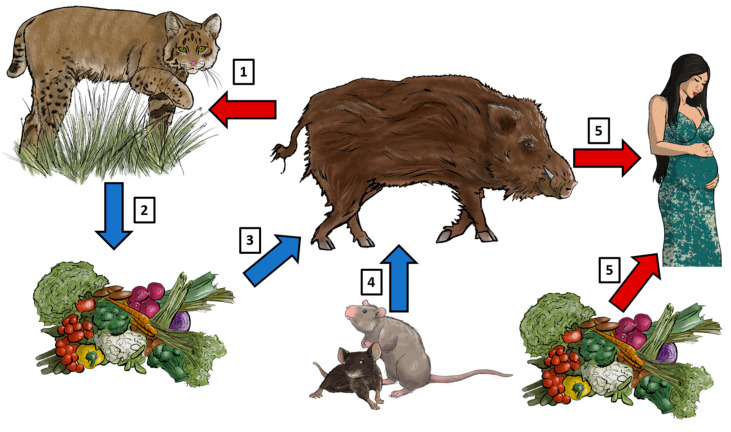
The wild boar as host and source of *T. gondii* infection. (1) Wild boars can become infected by oral ingestion of sporulated oocysts which are shed by feline species (i.e., wild cats); likewise, predation of infected wild boars can become a source of infection for cats. (2) Vegetables, crops, soil, and water can become contaminated with sporulated *T. gondii* oocysts. (3) Consequently, wild boars will ingest these sources of food and water and thus become infected with *T. gondii*. (4) Another route of infection for wild boars is the ingestion of small mammals infected with *T. gondii*. (5) Finally, human consumption of undercooked meat from *T. gondii*-infected wild boars can cause congenital toxoplasmosis in pregnant patients; likewise, vegetables contaminated with oocysts can also be a source of infection for humans. Blue arrows: source of *T. gondii* infection for the wild boar. Red arrows: infected wild boars as source of infection for humans and felines. Illustrations: Aleida Rentería.

Wild boars can be infected through *T. gondii* in different routes (Figure 1). They can accidentally ingest sporulated oocysts through contaminated water, soil, or food sources [19,20]. Moreover, intake of bradyzoite cysts can happen by predation or ingestion of infected prey, for example, small rodents. Wild boars can also play a role in keeping the *T. gondii* cycle by acting as source of infection to the final host (e.g., *Felis silvestris*) [21] (Figure 1).

In Central Europe, *T. gondii* seroprevalence in wild boars is reported between 16.80% to 48.00% [22,23,24,25]. With the highest percentage of seropositive animals found in Poland (48.00%) [25]. In Germany, a prevalence of *T. gondii* antibodies in wild boars has been reported only in animals from the Federal state of Brandenburg [22,26]. The objective of this investigation is to expand the current knowledge by reporting the seroprevalence of *T*. *gondii* in free-ranging wild boars from the Federal State of Thuringia, in central Germany.

## 2. Materials and Methods

### 2.1. Area of Study and Sample Collection

**Figure 2:**
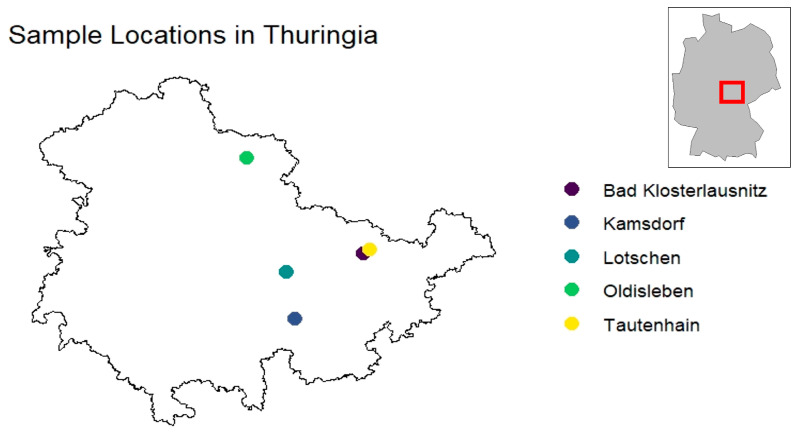
Collection areas in the Federal State of Thuringia.

Legally hunted animals were collected in the Federal State of Thuringia, in central Germany, from December 2017 to February 2018. The animals were from 5 municipalities in the eastern (Bad Klosterlausnitz, Lotschen, Tautenhain), southern (Kamsdorf), and the northern part of the state (Oldisleben) (Figure 2). The landscape of these areas consists of mixed forests and rural regions with human recreational activities. Cardiac blood was collected from the animals immediately after they were shot during hunts and stored at 4 °C until further investigation. Centrifugation was performed within 48 h (h) at 1600× *g* and 4 °C for 10 min. Subsequently, the serum samples were stored at −80 °C.

### 2.2. Serological Testing

Antibodies against *T. gondii* were detected in serum using the ID Screen^®^ Toxoplasmosis Indirect Multi-species (ID.Vet Innovative diagnostics, Grabels, France) according to the manufacturer’s instructions. Optical densities (OD_450mm_) were measured using a microplate reader 800 TS (Biotek Instruments, Winooski, VT, USA). The results were presented as S/P ratios using the following formula provided by the kit’s manufacturer:

S/P % = [(OD_sample_ − OD_negative control_)/(OD_positive control_ − OD_negative control_)] × 100

The interpretation of the results was also conducted according to the manufacturer’s guidelines whereby samples with an S/P ratio ≤ 40% were considered negative, samples with an S/P ratio higher than 40% but lower than 50% were registered as doubtful, and samples with an S/P ratio ≥ 50% were positive.

### 2.3. Statistical Analysis

Data were presented as percentages (%) with their respective confidence intervals (95% CI). Seroprevalence was compared with wild boars’ collection area (municipalities) and age, using the Fisher’s exact tests (α = 0.05). Statistical analysis and data visualisation were performed in R (version 4.3.2, Vienna, Austria) [27] using the RStudio environment (Boston, MA, USA) [28].

## 3. Results

In total, sera from 42 wild boars were collected. Most of the animals were from Bad Klosterlautsnitz (*n* = 20) and only a single serum sample was from Tautenhain. The distribution of sample origins to the individual locations is shown in Table 1. Twenty-nine of the samples were juveniles (animals younger than 1 year of age), six animals were yearlings (between 1 and 2 years old), and seven were adults (older than 2 years).

**Figure 3:**
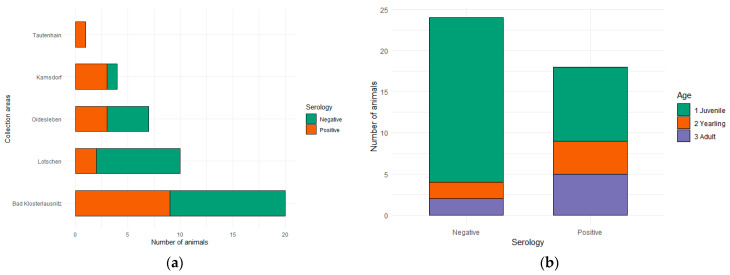
(**a**) Seroprevalence of wild boars across the different municipalities sampled in this study; (**b**) serology results with age distribution.

Overall, *T*. *gondii* antibodies (Immunoglobulin G, IgG) were successfully detected in 18 serum samples (37.50%, CI: 25.19–51.67%). In Bad Klosterlausnitz, 45.00% of the samples were positive (CI: 25.81–51.67%). The rest of the municipalities showed prevalences between 20.00% and 75.00%, with the exception of Tautenhain, where the only sample collected was positive (Table 1, Figure 3a). Seroprevalences per age were as follows: 31.03% (CI: 17.14–49.37%) in juveniles, 66.67% (CI: 29.57–90.75%) in yearlings, and 71.49% (CI: 35.24–92.44%) in adults (Table 1, Figure 3b). Seropositivity did not vary significantly across state districts (*p* = 0.25) or ages (*p* = 0.08) (Table 1). A summary of the results can be found in Table 1.

## 4. Discussion

We report for the first time the seroprevalence of *T. gondii* in free-ranging wild boars from Thuringia in central Germany. To the authors’ knowledge, two studies from the same research group in the north eastern part of the country precede our efforts [22,26]. In one of these studies, Bier et al. [22] reported seroprevalences of 24.14% and 25.00% in wild boars from the Federal State of Brandenburg, eastern Germany, during the hunting seasons of 2017/2018 and 2018/2019, respectively. We found a higher seroprevalence (37.50%) in the Thuringia animals during the same time point, namely the hunting season of 2017/2018; however, our sampled population is smaller than the one investigated in Brandenburg. A larger sample size, if possible, should be considered for future investigations in the area.

Spatial and temporal variations in parasite prevalence are not a rarity, even within the same country. This is particularly true for multi-host parasites like *T. gondii* [29]. One example of this can be found in Italy. Ranucci et al. [30] reported a seroprevalence of 14.00% in collected wild boars from the central part of the country. During the same time frame, 16.19% of the sampled wild boars in northern Italy were seropositive to *T. gondii* [31]. Around 10 years later in 2023, Villa et al. [32] reported a 53.10% seroprevalence in wild boars collected also in the north of Italy. Interestingly, another recent study from the Emilia-Romagna region (also northern Italy) reported 22.60% of seropositive animals [33].

In Germany, a less dramatic variation was found in Brandenburg. After Bier et al. showed the seroprevalences previously mentioned (24.14% and 25.00%) during the hunting seasons of 2017/2018 and 2018/2019 [22], the same group reported a seropositivity of 14.30% in wild boars collected in the exact same areas from Brandenburg during the following hunting seasons of 2019/2020 and 2020/2021 [26]. In our study, we only had access to samples from a single hunting season. However, given the previous record of temporal differences in *T. gondii* seropositivity in wild boars from Brandenburg, an expansion of the current dataset across later hunting seasons in Thuringia should be pursued.

In humans, the overall *Toxoplasma* seropositivity in Germany is higher than the global mean with around half of the adult population showing antibodies against *T. gondii* [34]. Moreover, a difference between the eastern and western part of Germany seems evident, with the eastern area showing a significantly higher number of seropositive inhabitants than the western region [35]. The federal state of Thuringia is located in the eastern part of Germany. The collection of samples in a previous study in Germany took place in the federal state of Brandenburg [22,26], which is also situated in the eastern part of the country.

In Germany, one of the main factors associated with *T. gondii* infection in humans is high meat consumption [35]. Current meat hygiene regulations in Europe neglect *T. gondii* meat infection [4]. Moreover, they focus on the detection of macroscopic findings such as Cysticerci in tissue, nematode larvae or young *Fasciola* spp. or *Ascaris suum* larvae migration lesions in some organs such as the liver. Additional tests include the official *Trichinella* meat inspection which can help to identify not only *Trichinella* spp. larvae but also *Alaria alata* mesocercariae [4]. However, none of these techniques can be used for the diagnosis of *T. gondii*. Approaches such as PCR or real-time polymerase chain reaction (PCR) or serological tests, for example, ELISA, can identify *T. gondii* antibodies in meat [26]. However, their use in routine meat hygiene means is cost and time intensive and requires trained personnel. Instead of this, simple common practices such as proper cooking of meat between 60 °C and 70 °C or freezing the game at −20 °C for at least 3 days can inactivate the parasite, and thus make the meat safe for human consumption [36].

In order to increase the efforts to educate the public regarding the proper way to handle and cook meat to inactivate *T. gondii*, efforts need to be first directed to elucidate the prevalence of *T. gondii* in game. The information currently available is insufficient. We hope that our study can attract more investigations like ours and, thus, increase the database available to accurately evaluate the risk game meat consumption possesses for the general population. This could also help to enhance strategies and policies to inform the consumer about the urgency of properly handling game meat in order to inactivate *T. gondii*.

## 5. Conclusions

The present study is a presentation of *T. gondii* seropositivity in free-ranging wild boars from Thuringia, central Germany. The seroprevalence in this small database is higher than the previously reported data in another region in the country. Nevertheless, the reduced sample size of our study should be taken into consideration before drawing strong conclusions. We highlight the importance of further studies with a larger number of animals and a country-wide investigation aimed to explicate the prevalence, and its possible variations, of *T. gondii* in wild boars across Germany. With this, a risk analysis program could be developed. This is of particular importance for sectors of the population who regularly consume wild boar meat: hunters, their inner circle and other game meat enthusiasts. We also hope to bring *T. gondii* into the spotlight as a present but sometimes neglected meat borne parasite.

## Figures and Tables

**Table 1:** Seroprevalences by collection area and age.

Parameter	Group	Positive/Total ^1^	Percentage (%) of Positives (95% CI)	*p*-Value
Total of tested animals		18/42	37.50 (25.19–51.67)	
Collection area	Bad Klosterlausnitz	9/20	45.00 (25.81–65.81)	0.25
(Municipality)	Kamsdorf	3/4	75.00 (28.91–96.59)	
	Lotschen	2/10	20.00 (4.59–52.06)	
	Oldisleben	3/7	42.86 (15.75–75.02)	
	Tautenhain	1/1	100.00 (16.75–100.00)	
Age	Juvenile ^2^	9/29	31.03 (17.14–49.37)	0.08
	Yearling ^2^	4/6	66.67 (29.57–90.75)	
	Adult ^2^	5/7	71.43 (35.24–92.44)	

^1^ Number of seropositive samples/Total number of samples tested in this group. ^2^ Juvenile: animals less than a year old; yearling: animals between one and two years of age; adult: animals older than two years.

## Data Availability

Data are contained within the article.
